# Mixed tumor of the vagina with atypical features: a case report including clinical management

**DOI:** 10.1016/j.gore.2026.102111

**Published:** 2026-05-20

**Authors:** Natalie Celestino, Amma Asare, Anais Malpica, Debby Rampisela, Jamie Dockery, Shrina Patel, Pamela Soliman, Travis T. Sims

**Affiliations:** a McGovern Medical School at UTHealth Houston, Houston, TX, USA; b Department of Gynecologic Oncology and Reproductive Medicine, The University of Texas MD Anderson Cancer Center, Houston, TX, USA; c Department of Pathology, The University of Texas MD Anderson Cancer Center, Houston, TX, USA; d Department of Pathology, Baylor Scott and White Medical Center–Temple, Temple, TX, USA

**Keywords:** Mixed vaginal tumor, Rare vaginal tumor, Pleomorphic adenoma

## Abstract

**Objectives:**

To present a rare case of mixed vaginal tumor with atypical features and to discuss clinical management in the context of uncertainty regarding biological behavior.

**Methods:**

We present the clinical course of a 50-year-old woman with a history of endometriosis and a recurrent vaginal mass who was diagnosed with a mixed tumor with atypical features and uncertain behavior. Following wide local excision, the multidisciplinary team reached a consensus to proceed with close surveillance without initiating adjuvant therapy.

**Results:**

The patient underwent excision of a 1 × 2 cm pedunculated vaginal mass with 1–2 cm margins. Pathology confirmed a mixed tumor with atypical features, 13 mitoses per 10 high-power fields, and irregular borders. Postoperatively, the patient had episodes of severe vaginal pain and swelling, including foul-smelling discharge and dysuria at day 9, and recurrent pain at 1 and 2 months. Imaging demonstrated only mild postsurgical changes without abscess, and symptoms resolved with antibiotics and supportive care. Given the absence of high-risk pathological features and no evidence supporting adjuvant therapy, the multidisciplinary team recommended surveillance. Three months after resection, the patient remained without evidence of disease.

**Conclusions:**

This case underscores the need for greater awareness and research to guide management and recurrence prevention for rare vaginal tumors. Complete resection is critical, and the potential benefits of adjuvant therapy must be weighed against risks such as sexual dysfunction, psychological impact, and treatment-related morbidity.

## Highlights

• Treatment of rare mixed vaginal tumor with atypical features and uncertain malignant potential.
• Wide local excision achieved mostly negative margins with minimal residual concern.
• Multidisciplinary team favored surveillance over adjuvant therapy due to absence of high-risk pathology.
• Management of rare vaginal tumors remains difficult due to scarce evidence, underscoring the need for further investigation.
• Long-term follow up remains critical in such cases as the recurrence risk and behavior remains poorly defined.

## 1 Introduction

Benign mixed tumors of the vagina are rare with the first reported case by Brown in 1953, describing a distinctive neoplasm characterized by an admixture of epithelial and mesenchymal components (Oliva et al., 2004). Since then, mixed tumors have been documented in various anatomical sites including the breast, mediastinum, and trachea (Mahe et al., 2012). Within the female genital tract, these lesions are also known as spindle cell epithelioma and are characteristically composed of epithelial and stromal cells in varying proportions (Oliva et al., 2004, Branton and Tavassoli, 1993, Wright et al., 1991, Kang and Yoon, 2002, Murdoch et al., 2003, Song et al., 2017, Tang et al., 2025).

Fewer than 60 cases of mixed tumor of the vagina are reported in the literature (Oliva et al., 2004); and only a small subset, fewer than 10 cases, has been described with atypical histologic features. These tumors typically occur in women aged 40–50 years of age (Oliva et al., 2004) and most commonly arise in the lower third of the vagina near the hymenal ring, a location common for benign polyps and cysts, which frequently leads to misdiagnosis (Oliva et al., 2004, Mahe et al., 2012). Clinically, these lesions present as nodular masses, with most patients being asymptomatic at presentation (Laskin et al., 2001).

Histologically, vaginal mixed tumors demonstrate a complex architecture with both epithelial and mesenchymal components in varying proportions (Oliva et al., 2004). Unlike salivary gland mixed tumors, vaginal mixed tumors lack the dual epithelial-myoepithelial combination morphology of their salivary gland counterparts (Fukunaga et al., 1996). Their immunohistochemical profile is distinctive, demonstrating coexpression of epithelial and mesenchymal markers (Oliva et al., 2004, Skelton and Smith, 2001). Reported studies show positivity for cytokeratin (AE1/3, CK7) in 67–92% of cases, muscle actin in 92%, desmin in 91%, and extensive CD10 expression in 100% of cases (Oliva et al., 2004). The diffuse expression of CD34, CD99, and Bcl-2, combined with negativity for S-100 protein, supports origin from a primitive pluripotential cell rather than myoepithelial differentiation (Oliva et al., 2004, Skelton and Smith, 2001). Furthermore, strong expression of estrogen and progesterone receptors suggests hormonal responsiveness and Müllerian derivation (Mahe et al., 2012, Branton and Tavassoli, 1993, Murdoch et al., 2003, Skelton and Smith, 2001).

While most vaginal mixed tumors are benign with good prognosis following simple excision, there also exist mixed vaginal tumors with atypical features. Atypical features that have been documented include increased mitotic activity, cellular atypia, mucoid degeneration with micro cyst formation and unusual architectural patterns (Kang and Yoon, 2002, Tang et al., 2025). Although rare, recurrence has been documented even in benign mixed tumors, particularly in larger lesions, emphasizing the importance of complete excision and long-term follow-up (Wright et al., 1991).

We present a case of mixed tumor of the vagina with atypical features, contributing to the limited literature on this rare entity and highlighting the diagnostic challenges posed by morphological variants of this uncommon neoplasm.

## 2 Case description

**Fig. 1:**
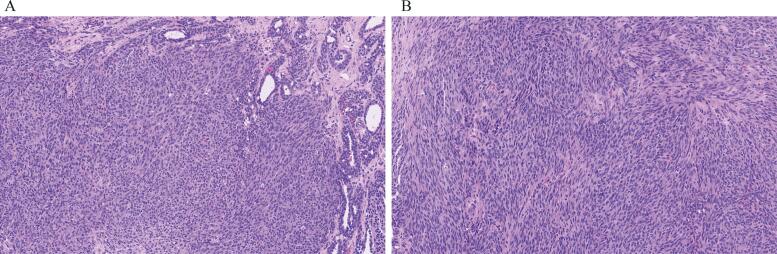
Vaginal mixed tumor, a proliferation of bland spindle cells intermixed with glandular elements, numerous mitoses in the spindle cells (A), spindle cells with numerous mitoses (B), hematoxylin and eosin, 20 x.

**Fig. 2:**
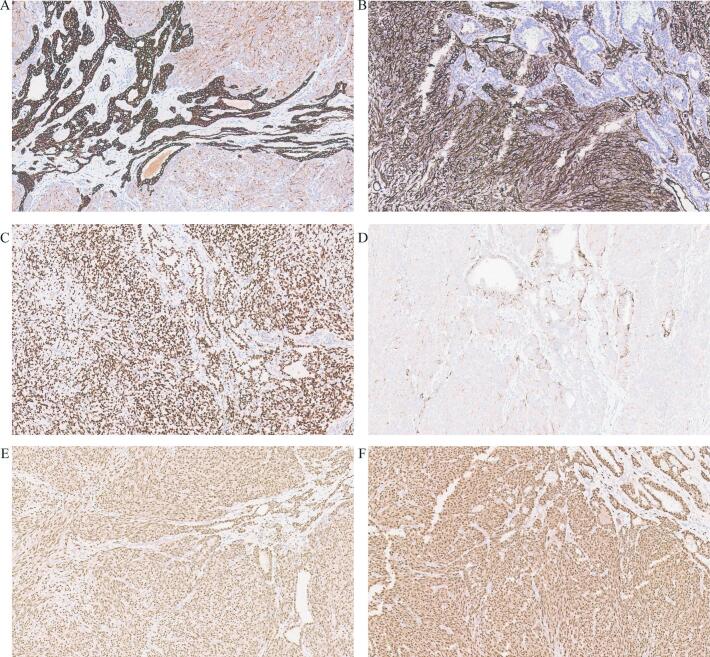
Immunohistochemical staining for keratin AE1/ AE3 (A), CD34 (B), WT-1 (C), desmin (D), estrogen receptor (E), and progesterone receptor (F), 20x.

A 50-year-old woman, status post-hysterectomy and with a history of endometriosis, reported routine cervical cancer screening, though specific cytology and HPV results were not available for review, presented to her gynecologist with a right vaginal wall, painful mass. Physical examination showed that the lesion was in the outer vaginal wall at the level of the urethra without communicating with it. The mass, clinically favored to be a cyst, persisted after treatment with antibiotics; therefore, it was excised. The surgical specimen was a 2.0 × 1.8 × 1.5 cm disrupted portion of rubbery, tan, white-tissue with no cyst contents. Its microscopic examination showed a solid proliferation of bland, plump, spindle cells admixed with glandular elements lined by low columnar and cuboidal cells with no atypia. The spindle cell component showed up to 18 mitoses per 10 high power fields (HPFs) (Fig. 1A and 1B). Immunohistochemically, the tumor cells were positive for AE1/AE3, CK 7 (focally), desmin, CD34, CD10, WT-1, ER (100%, strong), and PR (100%, strong) while negative or essentially negative for PAX8, p40, p63, caldesmon, SMA, calponin, myogenin, inhibin, calretinin, SS18-SSX, GFAP, and S100; the expression of p53 was wild type, p16 was patchy positive and the Ki-67 proliferative index was elevated (approximately 60%.) (Fig. 2A to 2F). The tumor extended to the margins (Fig. 1A). Nearly 4 weeks post initial excision, the patient reported recurrence of the mass with worsening vaginal discomfort. Positron emission tomography (PET)/CT demonstrated an ^18^F-fluorodeoxyglucose (FDG)-avid lesion in the distal vagina with a suspicious FDG-avid right pelvic lymph node and probable reactive right axillary node, without evidence of distant metastases. Pelvic magnetic resonance imaging confirmed a 1.2 × 1.4 cm mass along the right anterolateral distal vaginal wall and an enlarged right common femoral/external iliac lymph node (2.1 × 1.7 cm) demonstrating increased FDG uptake. Results of interventional radiology–guided biopsy of the pelvic node were negative for malignancy.

**Fig. 3:**
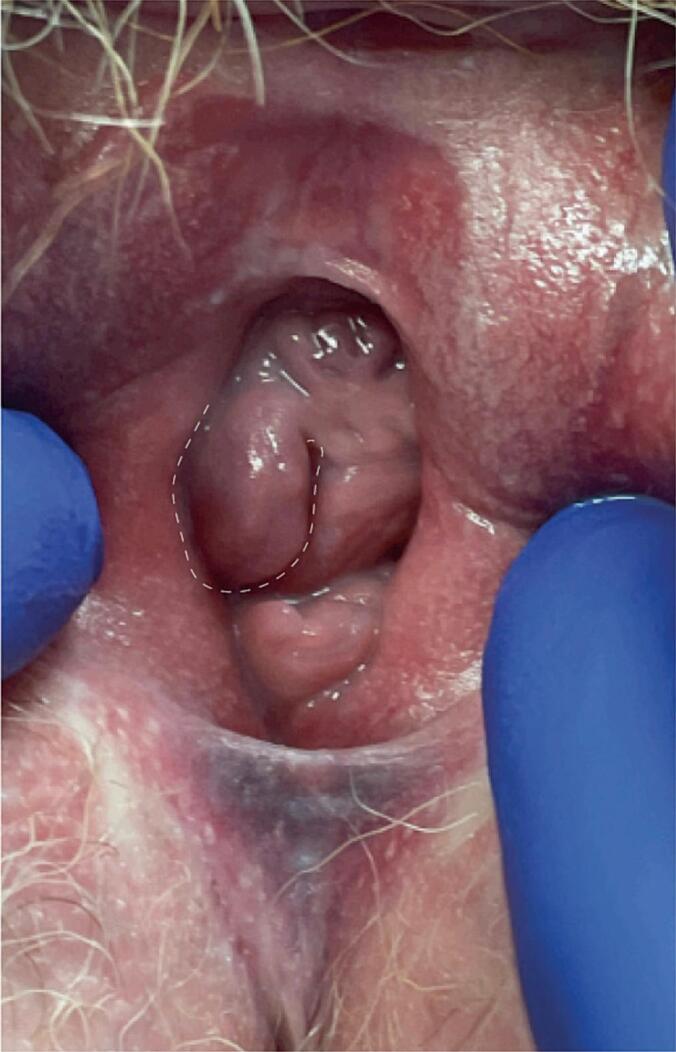
Recurrent vaginal mixed tumor prior to second wide local excision. The pedunculated lesion is noted with dashed white line.

**Fig. 4:**
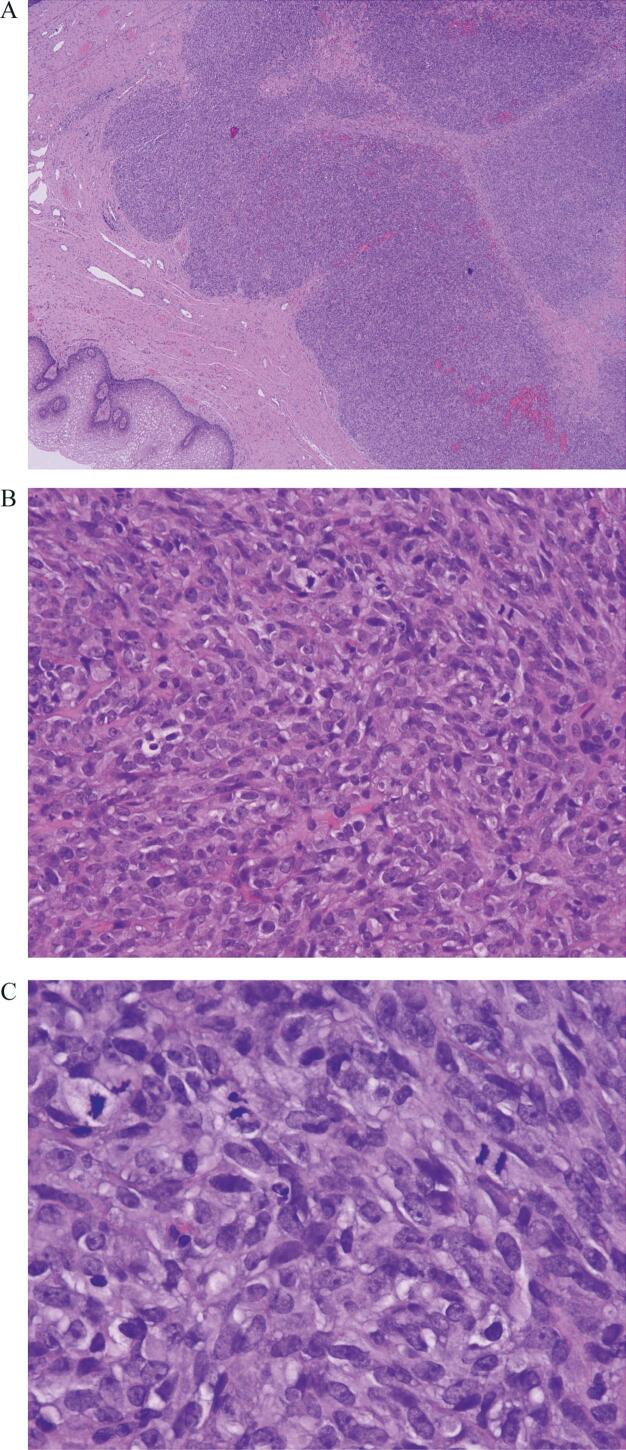
Vaginal mixed tumor in the wide local excision, showing irregular borders and only plump spindle cells, hematoxylin and eosin, 4× (A), plump spindle cells with atypia, hematoxylin and eosin, 20x (B), and 40× (C).

Eight weeks after the initial surgery, the patient underwent a wide local excision. The lesion was located at the site of the prior excision as a ∼ 1 cm pedunculated vaginal mass (Fig. 3). The surgical specimen measured 2.0 × 1.7 × 1.4 cm and contained a solid, tan-white lesion that measured 1.0 × 0.8 × 0.7 cm. Microscopic examination showed an irregular border, and a dense proliferation of plump, spindle cells with atypia, and 13 mitoses per 10 HPFs (Fig. 4A, 4B, 4C). Immunohistochemical studies showed that the tumor cells were positive for keratin (patchy), EMA (weak), ER (100%, strong), PR (100%, strong), WT-1, CD34, bcl2, and p16 (patchy) and focally positive for desmin and calponin. The tumor cells were negative for GFAP, p63, and S100. The expression of p53 was wild-type and BRG1 and INI-1 were retained. All margins were negative, but the inferior margin could not be assessed due to preanalitycal factors (ie, tissue folding and tangential sectioning). The presence of atypia, and increased mitotic index warranted the designation of mixed tumor with atypical features/transformation and uncertain behavior. Molecular testing including MDA MAPP (mutation analysis precision panel), and Mayo Complete Sarcoma Panel showed no mutations, in addition to a low tumor mutation burden (TMB: 1mut/MB), microsatellite stability (MSS), no fusions and a MED12 variant of uncertain significance [c.4130C > T (Exon 30), amino acid change:p.S1377F(Ser1377Phe), variant allele frequency:51.6%)]. Given the lack of evidence supporting adjuvant therapy for this patient, the multidisciplinary team recommended surveillance.

The patient’s postoperative course was complicated by a superficial wound infection. Nine days after surgery, she presented to the emergency department with severe vaginal pain, foul-smelling discharge, and dysuria and was treated with a 7-day course of doxycycline and metronidazole, which was followed by symptom resolution. A little over 1 month later, she returned with swelling and pain at the surgical site; CT with contrast demonstrated mild nonspecific enhancement and thickening of the right vulvo-vaginal region consistent with postsurgical change, without drainable collection, and interval reduction of right inguinal lymphadenopathy. Nearly 2 months postoperatively, she presented again with pelvic pain and a newly perceived vaginal mass; CT abdomen and pelvis with contrast findings were negative, no mass was perceived on physical exam and symptoms subsequently resolved. Eleven months after the second resection, the patient remained without evidence of disease.

## 3 Discussion

Due to the rarity of mixed tumors of the vagina, evidence guiding management is derived almost exclusively from individual case reports and small case series (Oliva et al., 2004, Mahe et al., 2012, Branton and Tavassoli, 1993, Wright et al., 1991, Kang and Yoon, 2002, Murdoch et al., 2003, Song et al., 2017, Tang et al., 2025). Despite this limitation, local excision has been the mainstay of treatment for mixed tumors of the vagina dating back to 1981 and has been shown to have excellent outcomes (Oliva et al., 2004, Kang and Yoon, 2002). In the present case, the initial lesion was clinically described as a 1.5 cm periurethral vaginal cyst and appeared well circumscribed at the time of surgery, leading to a local excision with the mass shelled out from the surrounding tissue. Given its small size, benign cystic appearance, and proximity to periurethral structures, a more extensive resection was not initially pursued. Gross intraoperative assessment of margins in these lesions may be challenging, as tumors often appear well circumscribed clinically. Recurrence is rare but when observed has been attributed to incomplete excision of the primary lesion rather than aggressive tumor biology (Oliva et al., 2004, Branton and Tavassoli, 1993, Wright et al., 1991, Kang and Yoon, 2002). These findings support surgical management of all rare vaginal tumors, including those describing variable or atypical histologic features (Kang and Yoon, 2002, Murdoch et al., 2003, Sirota et al., 1981).

There is little published evidence regarding the size of a negative margin with recurrence and prognosis. Importantly, there is also no clear evidence to support the use of adjuvant therapy or chemotherapy for benign or atypical mixed tumors of the vagina broadly. Across published reports, patients were often treated with excision alone, and no benefit of adjuvant therapy has been described (Kang and Yoon, 2002, Murdoch et al., 2003, Song et al., 2017, Sirota et al., 1981, van den Broek et al., 1996). The absence of documented metastatic behavior, malignant transformation, or recurrence following complete excision further supports this approach. While close clinical follow up is recommended due to the rarity of these tumors, recurrence appears exceedingly uncommon and surgical excision with the attainment of negative margins remains appropriate (van den Broek et al., 1996).

For the present case, the multidisciplinary tumor board recommended surveillance after wide local excision, given the absence of high-risk pathological features, no evidence of metastasis, and the lack of evidence supporting adjuvant therapy in atypical mixed tumors of the vagina. After surgery, the patient underwent surveillance with serial office visits and pelvic examinations, including speculum and rectovaginal examinations. Follow-up occurred every 3 months initially and was later extended to 4-month intervals given no evidence of recurrence.

## 4 Conclusion

This case illustrates a rare vaginal mixed tumor with atypical features and uncertain behavior. The patient remained disease-free after surgical excision and remained under close surveillance at last follow-up. The rarity of this tumor limits evidence-based management guidelines, highlighting the importance of reporting cases to improve understanding. Complete excision in such cases is critical, and the decision for adjuvant therapy should be individualized to balance recurrence risk against quality-of-life considerations and adverse effects.

## 5 Consent

Written informed consent was obtained from the patient for publication of this case report and accompanying images. A copy of the written consent is available for review upon request.

## CRediT authorship contribution statement

**Natalie Celestino:** Writing – review & editing, Writing – original draft. **Amma Asare:** Writing – review & editing, Writing – original draft, Supervision, Data curation, Conceptualization. **Anais Malpica:** Writing – review & editing, Visualization, Supervision. **Debby Rampisela:** Investigation, Formal analysis. **Jamie Dockery:** Writing – review & editing, Project administration. **Shrina Patel:** Writing – review & editing, Supervision. **Pamela Soliman:** Writing – review & editing. **Travis T. Sims:** Writing – review & editing, Writing – original draft, Supervision, Project administration, Formal analysis, Conceptualization.

## Declaration of competing interest

The authors declare that they have no known competing financial interests or personal relationships that could have appeared to influence the work reported in this paper.
